# Surgical Effects of Resecting Skull Base Tumors Using Pre-operative Multimodal Image Fusion Technology: A Retrospective Study

**DOI:** 10.3389/fneur.2022.895638

**Published:** 2022-05-12

**Authors:** Zhi-heng Jian, Jia-yan Li, Kai-hua Wu, Yu Li, Shi-xue Li, Hai-dong Chen, Gang Chen

**Affiliations:** ^1^Department of Neurosurgery, Zhuhai People's Hospital (Zhuhai Hospital Affiliated With Jinan University, China), Zhuhai, China; ^2^Department of Radiology, Zhuhai's People Hospital, Zhuhai, China

**Keywords:** image infusion, skull base tumor, cerebral fluid leakage, skull base reconstruction, micro-surgical procedure, neurosurgery

## Abstract

**Objectives:**

To analyze the surgical effects of resecting skull base tumors using multimodal three-dimensional (3D) image fusion technology in the neurosurgery department and present some typical cases.

**Methods:**

From October 2019 to October 2021, we included 47 consecutive patients with skull base tumors in the Neurosurgery Department at Zhuhai People's Hospital in this study. Pre-operative head computed tomography and magnetic resonance imaging data acquisition was performed using the GE AW workstation software for registration fusion, image fusion, and 3D reconstruction. The surgical approach and surgical plan were designed based on the multimodal 3D image, and the resection rate, complication rate, and operative time of the surgery using the multimodal image fusion technique were analyzed.

**Results:**

The reconstructed multimodal 3D images precisely demonstrated the size, location, and shape of the tumor along with the anatomical relationship between the tumor and surrounding structures, which is consistent with the intraoperative findings. Among 47 patients, 39 patients (78.7%) underwent total resection, 5 (14.9%) underwent subtotal resection, and 3 (6.4%) underwent partial resection. The mean operative time was 4.42 ± 1.32 h. No patient died during the inpatient period. Post-operative complications included 6 cases of cerebrospinal fluid leakage (14.9%), 3 cases of intracranial infection (6.4%), 6 cases of facial paralysis (12.8%), 2 cases of dysphagia (4.3%), and 1 case of diplopia (2.1%), all of which were improved after symptomatic treatment. The application value of pre-operative 3D image fusion technology was evaluated as outstanding in 40 cases (85.1%) and valuable in 7 cases (14.9%).

**Conclusions:**

Pre-operative multimodal image fusion technology can provide valuable visual information in skull base tumor surgery and help neurosurgeons design the surgical incision, choose a more rational surgical approach, and precisely resect the tumor. The multimodal image fusion technique should be strongly recommended for skull base tumor surgery.

## Introduction

Skull base tumors are a spectrum of tumor deriving from bone structure, dura mater, brain tissue, and outer cranial tissue adjoin with the skull base. They also have a large number of branching vessels involved in the blood supply, so intraoperative bleeding can easily cause an unclear surgical field (1–3). Moreover, some skull base tumors are accompanied by destruction of the skull base bone structure (4, 5). The total resection rate varies from 57 to 77% (6–10). Skull base tumor surgery requires a high order of neuroanatomical knowledge and a substantial surgical experience; therefore, it has been recognized as one of the most challenging types of neurosurgery. Yet, single-mode imaging has been unable to meet the soaring demand of microinvasion and precise resection in neurosurgery, especially in skull base tumor resection. Multimodal image fusion is a three-dimensional (3D) reconstruction of multiple image data on the same image that provides a clear, intuitive, and overall display of the tumor and its spatial relationship with peripheral vessels, nerves, and brain tissue, as well as the skull structure related to the surgical approach. By applying to this technique, neurosurgeons can design a more rationale surgical approach to skull base tumor surgery, avoid unexpected injury, better protect the normal skull structure, and reduce complications (11–14). This technology was introduced in our department in October 2019. The present study aimed to analyze the surgical effects of resecting skull base tumors using multimodal 3D image fusion technology in the neurosurgery department and present some typical cases.

## Materials and Methods

### Ethics Statements

This study was approved by the Medical Ethics Committee of Zhuhai People's Hospital (Number: ZY.no20201001B06011231).

### Study Design and Patient Population

In total, 47 consecutive patients with skull base tumors who were admitted to the Neurosurgery Department of Zhuhai People's Hospital underwent microcraniotomy from October 2019 to September 2021 and were screened for the present retrospective study.

### Image Data Collection and Examination Methods

A computed tomography (CT) examination was performed using a GE Revolution CT (128-row) scanner (Boston, MA, USA) with a scan thickness of 1 mm. A Philips 3.0T Achieva TX MR scanner (Amsterdam, the Netherlands) was used for the magnetic resonance imaging (MRI) examination, and the imaging sequence included T1-weighted imaging (T1WI), T1WI 3D turbo field echo, T2-weighted imaging 3D fluid-attenuated inversion recovery (FLAIR), 3D phase contrast angiography magnetic resonance (MR) venography (MRV), contrast-enhance magnetic resonance angiography (MRA), 3D time of flight MRA, etc.

### Image Acquisition and Multimodal Image Fusion

CT and MR image data (Digital Imaging and Communications in Medicine format) of all patients were downloaded from the HIS information system, and then the image data were uploaded to the GE AW workstation. Corresponding image sequences were opened in the workstation to complete registration fusion and 3D reconstruction. Automatic registration and manual registration are used for registration. Automatic alignment imports required image data such as MR sequences and CT data, and then the Infusion function was selected for automatic matching. Manual registration uses characteristic anatomical markers (e.g., the center of the eyeball, pituitary fossa, sinuses, mastoid apex, digastric sulcus apex, etc.) as reference points. Three to five reference points were used for each case. The Smart Brush function was used to draw the outline of the tumor, and then the MRA, MRV, and other images were fused with the tumor to form the 3D digital anatomical image of the tumor and vessels. Finally, the 3D reconstruction image of the skull was added to the 3D image of the tumor with blood vessels by CT to complete the multimodal image fusion.

### Surgical Treatment Assisted by Multimodal Image Fusion Technology

**Figure 1:**
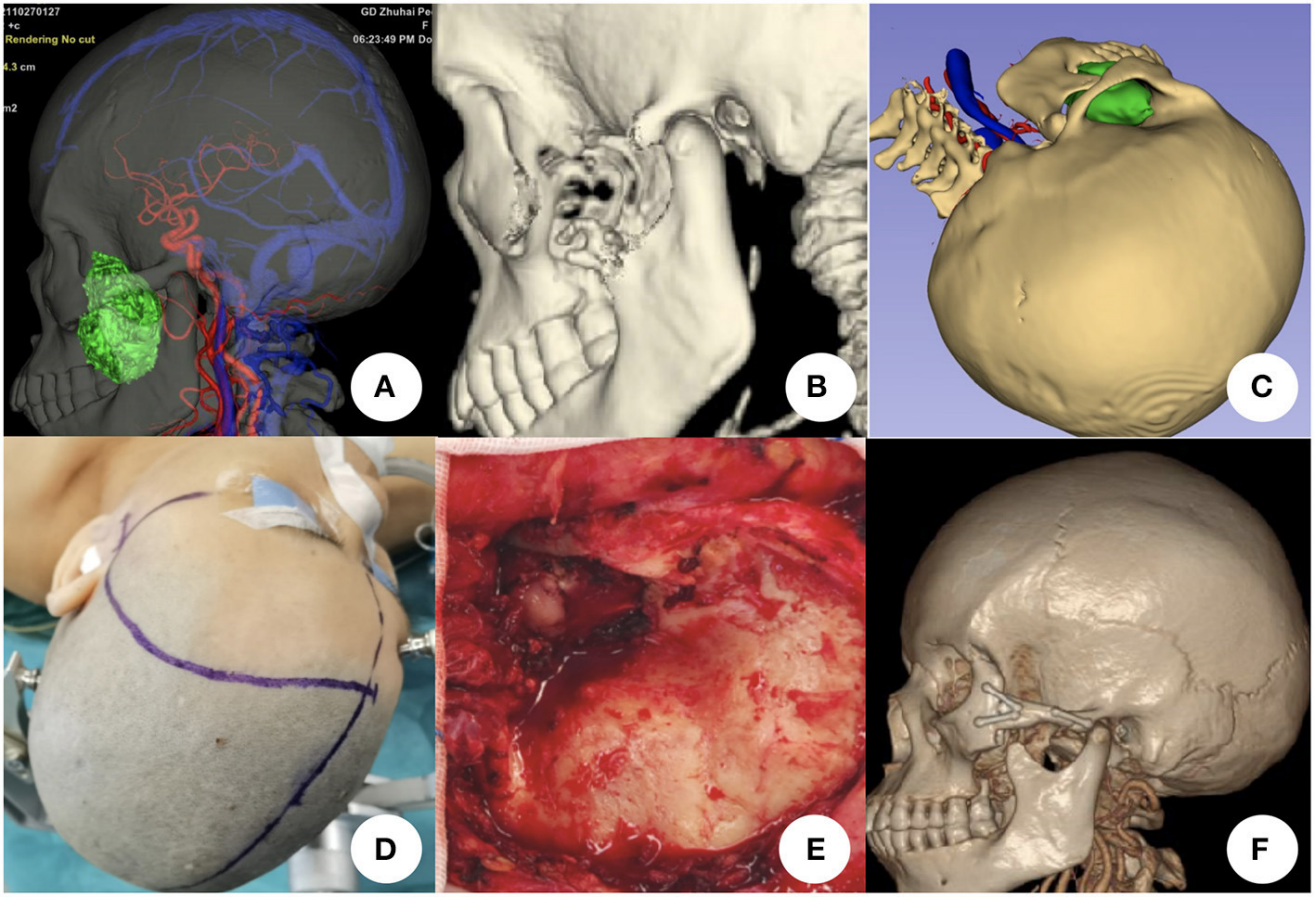
Surgical plan for resecting skull base tumors using multimodal image fusion technology. **(A)** Tumor in the left infra-temporal fossa shown by multimodal image fusion [combined with head computed tomography (CT), magnetic resonance imaging, magnetic resonance angiography, and magnetic resonance venography]. **(B)** Bony defect is demonstrated by head CT three-dimensional (3D) reconstruction. **(C)** Simulation of the surgical plan using multimodal fusion and 3D images. **(D)** Incision design based on simulated approach. **(E)** Tumor exposure and resection during the surgical procedure; the zygomatic arch is removed. **(F)** Post-operative head CT scan and 3D reconstruction showing that the tumor was totally resected and the zygomatic arch was reconstructed.

All patients were operated by one senior surgeon. Multimodal image fusion and 3D reconstruction were used to demonstrate the spatial relationship of the tumor and surrounding structures (Figure 1A). Specific details such as bony defection was evaluated (Figure 1B). Approach stimulation was performed based on the 3D reconstructed image (Figure 1C). The incision design was based on the stimulation result (Figure 1D). Intraoperative resection was performed along the tumor boundary, and attention was paid to protect the tumor and important tissues, such as the surrounding arteries, veins, and nerves, to achieve the maximum possible total tumor resection. For tumors invading the cavernous sinus, internal carotid artery, cranial nerves, and other tissues, subtotal resection or partial resection can be performed, and stereotactic radiotherapy should be performed after to detect residual tumors (Figure 1E). The post-operative image was used to evaluate the surgical effect (Figure 1F).

### Evaluation Criteria of the Application Value

The evaluation method of the application value of multimodal image fusion technology was based on Oishi et al.'s (15) study. All patients were operated by one senior surgeon and the overall application value was estimated by the surgeon after surgery. The evaluation grade was divided into three levels:

(1) Prominent: pre-operative multimodal image fusion technology is important to completion of the operation, and the expected surgical effect cannot be achieved by using single modal images only;
(2) Supportive: a single modal image can also achieve the expected surgical effect, but multimodal image fusion technology can provide a clearer and more intuitive demonstration of fine structures, which can reduce the injury of surgical collateral and benefit surgery; and
(3) No value: surgery can be completed as expected through using of a single modal image only, without the assistance of multimodal image fusion technology.

The evaluation criteria were as follows:

(1) whether the choice of surgical approach was appropriate;
(2) whether the judgment of spatial location of tumor was accurate;
(3) whether the location and adjacent relationship of vessels were accurate;
(4) whether the adjacent relationship between the tumor and venous sinus was accurate;
(5) whether the exposure of bone window was sufficient; and
(6) whether the surgical procedure was consistent with the pre-operative judgment.

### Surgical Resection Rate, Post-operative Complication Rate, and Follow-Up

#### Surgical Resection Rate

The total resection rate was determined based on the following brain MR examination results within 1–3 days after the operation (1):

(1) total resection (100% tumor resection with no radiographic residue);
(2) subtotal resection (more than 90% tumor volume reduction); and
(3) partial resection (0–90% tumor volume reduction) and unresection.

#### Complication Rate

The incidence of surgical complications during the perioperative period included olfactory disorders, blurred vision, protruding eyes, epistaxis, nasal obstruction, hearing loss, facial numbness, facial paralysis, eyelid closure, limb sensory, and limb motor disorders.

#### Operative Time

The operative time was estimated from the incision of the scalp to completion of tumor resection. The overall mean operative time was measured and analyzed.

#### Follow-Up

Patients were followed up within 3–12 months after surgery. Brain MR enhancement was reviewed to evaluate whether the tumor recurred or increased and assess whether there are new complications.

### Statistical Analysis

The *t*-test and χ^2^-test were used to compare the mean or frequency between the patient groups, respectively. Statistical analysis was performed using SPSS 25.0 (IBM Corp., Armonk, NY, USA). A *P*-value < 0.05 was considered to be statistically significant.

## Results

### Demographic and Clinical Characteristics

Forty-seven patients (19 men and 28 women) were included. Patients' average age was 51.11 ± 12.62 years (range, 12–77 years).

### Multimodal Image Fusion Outcome

**Figure 2:**
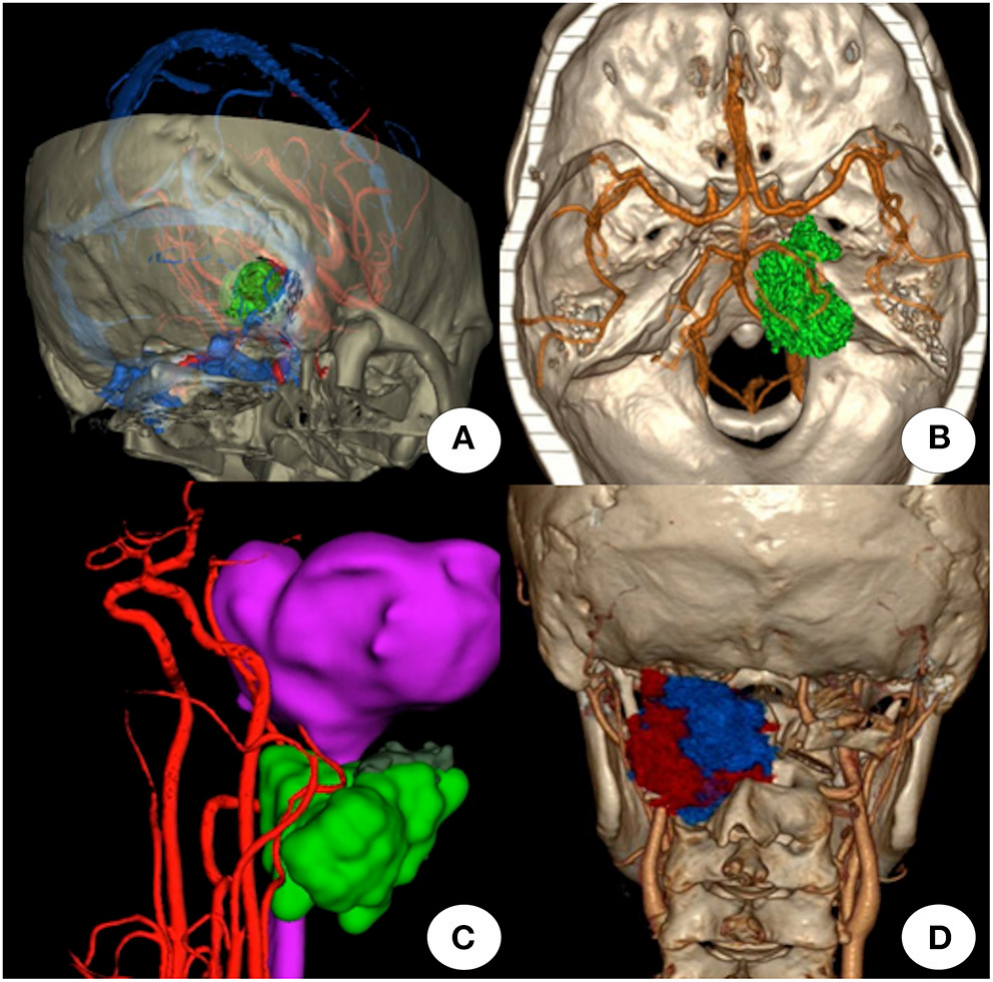
Effectiveness of multimodal image fusion technology. **(A)** Surgical plan of the retrosigmoid approach using a multimodal image fusion image (combined with head computed tomography, magnetic resonance imaging, magnetic resonance angiography, and magnetic resonance venography). **(B)** Multimodal image fusion demonstrates the spatial relationship of the tumor and surrounding structures. **(C)** Multimodal image fusion demonstrates the relationship between the left vertebral artery and tumor (green). **(D)** Multimodal image fusion demonstrates the spatial relationship between the tumor (red and blue), artery, and surrounding bony structures.

Pre-operative multimodal image fusion and reconstruction were completed in all 47 patients with skull base tumors. The 3D image after multimodal image fusion could be rotated arbitrarily, and the surface structure became transparent or translucent to show the internal structure of interest, when enabled design of the surgical approach and plan (Figure 2A). The spatial relationship of the tumor and surrounding structures (Figure 2B), spatial relationship of the vessels and lesions (Figure 2C), and demonstration of the intracranial communication lesion (Figure 2D) were all consistent with the intraoperative findings.

### Clinical Outcome

#### Tumor Characteristics

(1) Anterior skull base: There were left cranio-orbital communication tumors in 2 cases, olfactory groove/sphenoid platform meningiomas in 6 cases (left side, 3 cases; right side, 2 cases), and sphenoid ridge meningiomas in 3 cases (right side, 3 cases).
(2) Middle skull base: There were tumors in the saddle area/cavernous sinus area in 8 cases (left cavernous sinus area, 6 cases; right cavernous sinus area, 2 cases), temporal base/inferior temporal fossa in 6 cases (left side, 2 cases; right side, 4 cases), and petroclival area in 5 cases (left side, 4 cases; right side, 1 case).
(3) Posterior skull base: There were tumors in the cerebello-pontine angle (CPA) area in 10 cases (left side, 5 cases; right side, 5 cases), jugular foramen in 2 cases (left side, 2 cases), and occipitocervical junction in 4 cases (left side, 2 cases; right side, 2 cases).
(4) There were 14 cases of intracranial communication and 33 cases of non-intracranial communication. Recurrent tumors developed in 9 cases, and malignancy occurred in 6 cases. The volume of the mass was >3 cm^3^ in 32 cases (Table 1).

**Table 1:** Characteristic of the 47 cases of tumors.

**Position**	**Side**		**Communication**		**Tumor volume**		**Recurrence**		**Malignancy**	
	**Left**	**Right**	**Positive**	**Negative**	**>3 cm^**3**^**	**<3 cm^**3**^**	**Positive**	**Negative**	**Positive**	**Negative**
**Anterior**	**5**	**5**	**3**	**7**	**7**	**3**	**3**	**7**	**2**	**8**
Frontal-orbital area	2	0	2	0	1	1	1	1	1	4
Planum sphenoidale	3	2	1	4	4	1	1	4	1	4
Sphenoid ridge	0	3	0	3	2	1	1	2	0	3
**Middle**	**12**	**7**	**7**	**12**	**12**	**7**	**3**	**16**	**3**	**16**
Cavernous sinus	6	2	3	5	6	2	2	6	0	8
Infra-temporal fossa	2	4	3	3	3	3	1	5	3	3
Petroclival region	4	1	1	4	3	2	0	5	0	5
**Posterior**	**10**	**8**	**4**	**14**	**13**	**5**	**3**	**15**	**1**	**17**
CPA region	5	5	0	10	8	2	1	9	0	10
Jugular foramen	2	0	0	2	2	0	0	2	0	2
Occipital-cervical region	2	2	4	0	3	1	2	2	0	4
Brain stem	1	1	0	2	0	2	0	2	1	1
**Total**	**27**	**20**	**14**	**33**	**32**	**15**	**9**	**38**	**6**	**41**

*CPA, cerebello-pontine angle*.

#### Pathology of the Tumors

There were meningiomas in 23 cases, schwannomas in 14 cases, metastatic tumors in 3 cases, a spindle cell tumor in 1 case, mesenchymal malignant tumor in 1 case, giant cell granuloma in 1 case, cholesteatoma in 1 case, squamous cell carcinoma of the left external auditory canal with petrosal bone and temporal base infiltration in 1 case, pituitary adenoma in 1 case, and choroid plexus papilloma in 1 case (Table 2).

**Table 2:** Pathology of the tumors.

**Pathology**	**Position**			**Total**
	**Anterior**	**Middle**	**Posterior**	
Meningioma	8	12	3	23
Schwannoma	0	2	12	14
Metastasis	1	1	1	3
Spindle cell tumor	1	0	0	1
Cholesteatoma	0	0	1	1
Squamous carcinoma	0	1	0	1
Mesenchymal malignancy	0	1	0	1
Giant cell granuloma	0	1	0	1
Pituitary adenoma	0	1	0	1
Choroid plexus papilloma	0	0	1	1
**Total**	**10**	**19**	**18**	**47**

#### Outcome of Surgical Resection

Total resection was performed in 39 cases (39/47, 78.7%), subtotal resection in 5 cases (7/47, 14.9%), and partial resection in 3 cases (3/47, 6.4%). No perioperative deaths occurred. The cases of subtotal resection included a left trigeminal schwannoma in 1 case, right cavernous sinus meningioma in 1 case, breast cancer and brainstem metastasis in 1 case, and olfactory groove meningioma in 2 cases with ethmoid sinus infiltration in the maxillary sinus. Cases of partial resection included a left optic canal meningioma in 1 case, external auditory canal squamous cell carcinoma with left petrosal bone–temporal base infiltration in 1 case, and petroclival meningioma in 1 case. The differences of the tumor size, malignancy, recurrence and position of tumors were not statistical significant except the total resection rate of intracranial and extracranial communication tumors was lower than that of non-intracranial and extracranial communication tumors (71.4 and 87.9%, respectively, *P* = 0.001).

#### Operative Time

The mean operative time was 4.42 ± 1.32 h. The mean operative times in the anterior skull base group, middle skull base group, and posterior skull base group were 4.72 ± 0.98 h, 5.32 ± 1.28 h, and 3.92 ± 1.73 h, respectively. The differences of the tumor size, malignancy, recurrence and position of tumors were not statistical significant except the operation time of intracranial and extracranial communication tumors is longer than non-intracranial and extracranial communication tumors (5.72 ± 1.53 h and 4.12 ± 1.33 h, respectively, *P* = 0.012).

#### Complications

Six cases of cerebrospinal fluid leakage were cured by lumbar cistern drainage. Three cases of intracranial infection were treated with lumbar cistern drainage and antibiotic treatment. One case of intracranial hematoma was a meningioma of the anterior skull base, so a secondary operation was performed to evacuate the hematoma and decompress the bone flap. One case of infraction was an olfactory groove meningioma that was cured by conservative treatment. There were 9 cases of cranial nerve dysfunction, including 6 cases of facial paralysis, 2 cases of dysphagia, and 1 case of oculomotor nerve paralysis; 6 patients improved after symptomatic treatment and hormone shock therapy. Two patients with posterior cranial nerve paralysis underwent tracheotomy and recovered after discharge.

#### Follow-Up

Forty-five cases were successfully followed up after discharge, and 2 cases missed follow-up. The 45 patients were followed up for 3–12 months, with an average of 7 months. One patient with metastatic lung cancer died. Among 39 patients who underwent total resection of the tumor, 2 patients had recurrence after surgery, namely 1 patient with a malignant meningioma of the anterior skull base and 1 patient with a trigeminal schwannoma. There were no new complications in the remaining 44 cases except 1 mortality.

### Application Value of Multimodal Image Fusion Technology

Among 47 cases of skull base tumors, 40 cases were evaluated as prominent and 7 cases as supportive. The multimodal image fusion-assisted pre-operative approach was reasonable in all cases. The spatial location and surrounding tissue structure of the tumor reconstructed in all cases were consistent with the intraoperative findings. The reconstructed vessels, venous sinuses, and cranial nerves in all cases were consistent with the intraoperative findings. Two cases had overexposure of the bone window (Table 3).

**Table 3:** Summary of cases, pre-surgical plan, and surgical outcomes.

**Case no**.	**Location**	**Given evaluation**	**Spatial aspects of tumor**	**Arterial information**	**Venous information**	**Bone window**	**Surgical resection**	**Achievement**
1	Occipital-cervical region	Prominent	Satisfactory	Satisfactory	Satisfactory	Reasonable	GTR	As planned
2	Frontal-orbital area	Prominent	Extension to the bone	Satisfactory	Satisfactory	Reasonable	PR	Inefficient removal due to extension to the bone
3	Planum sphenoidale	Supportive	Satisfactory	Satisfactory	Satisfactory	Reasonable	STR	Inefficient removal due to bleeding
4	Cavernous sinus	Prominent	Satisfactory	Satisfactory	Satisfactory	Reasonable	GTR	As planned
5	Sphenoid ridge	Supportive	Satisfactory	Satisfactory	Satisfactory	Reasonable	GTR	As planned
6	Cavernous sinus	Prominent	Satisfactory	Satisfactory	Satisfactory	Reasonable	GTR	As planned
7	Infra-temporal fossa	Prominent	Satisfactory	Satisfactory	Satisfactory	Reasonable	GTR	As planned
8	Petroclival region	Prominent	Extension to the bone	Satisfactory	Satisfactory	Reasonable	PR	Inefficient removal due to bleeding
9	Cavernous sinus	Prominent	Satisfactory	Satisfactory	Satisfactory	Reasonable	STR	Inefficient removal due to brain swelling
10	CPA region	Supportive	Satisfactory	Satisfactory	Damage to the petrosal vein	Reasonable	GTR	As planned
11	Jugular foramen	Prominent	Satisfactory	Satisfactory	Satisfactory	Reasonable	GTR	As planned
12	Planum sphenoidale	Prominent	Satisfactory	Satisfactory	Satisfactory	Overexposure	STR	Inefficient removal due to brain swelling
13	Occipital-cervical region	Prominent	Satisfactory	Satisfactory	Satisfactory	Reasonable	GTR	As planned
14	Planum sphenoidale	Supportive	Satisfactory	Satisfactory	Satisfactory	Reasonable	GTR	As planned
15	Planum sphenoidale	Prominent	Satisfactory	Satisfactory	Satisfactory	Reasonable	GTR	As planned
16	Cavernous sinus	Supportive	Extension to the major artery	Unable to fully demonstrate the degree of adhesion	Satisfactory	Reasonable	STR	Inefficient removal due to location of ICA and tumor
17	Sphenoid ridge	Prominent	Satisfactory	Satisfactory	Satisfactory	Reasonable	GTR	As planned
18	Cavernous sinus	Prominent	Satisfactory	Satisfactory	Satisfactory	Reasonable	GTR	As planned
19	Infra-temporal fossa	Prominent	Extension to the bone	Satisfactory	Satisfactory	Reasonable	PR	Inefficient removal due to bleeding
20	Petroclival region	Prominent	Satisfactory	Satisfactory	Satisfactory	Reasonable	GTR	As planned
21	CPA region	Prominent	Satisfactory	Satisfactory	Satisfactory	Reasonable	GTR	As planned
22	CPA region	Prominent	Satisfactory	Satisfactory	Satisfactory	Reasonable	GTR	As planned
23	Jugular foramen	Prominent	Satisfactory	Satisfactory	Satisfactory	Reasonable	GTR	As planned
24	Occipital-cervical region	Prominent	Satisfactory	Satisfactory	Satisfactory	Reasonable	STR	Inefficient removal due to bleeding
25	Occipital-cervical region	Supportive	Satisfactory	Satisfactory	Satisfactory	Reasonable	GTR	As planned
26	CPA region	Supportive	Satisfactory	Satisfactory	Satisfactory	Reasonable	GTR	As planned
27	Frontal-orbital area	Prominent	Satisfactory	Satisfactory	Satisfactory	Reasonable	GTR	As planned
28	Planum sphenoidale	Prominent	Satisfactory	Satisfactory	Satisfactory	Reasonable	GTR	As planned
29	Cavernous sinus	Prominent	Satisfactory	Satisfactory	Satisfactory	Reasonable	GTR	As planned
30	CPA region	Prominent	Satisfactory	Satisfactory	Satisfactory	Reasonable	GTR	As planned
31	Cavernous sinus	Prominent	Satisfactory	Satisfactory	Satisfactory	Reasonable	GTR	As planned
32	Infra-temporal fossa	Prominent	Satisfactory	Satisfactory	Satisfactory	Reasonable	GTR	As planned
33	Petroclival region	Prominent	Satisfactory	Satisfactory	Satisfactory	Reasonable	GTR	As planned
34	Cavernous sinus	Prominent	Satisfactory	Satisfactory	Satisfactory	Reasonable	GTR	As planned
35	CPA region	Prominent	Satisfactory	Satisfactory	Satisfactory	Reasonable	GTR	As planned
36	CPA region	Prominent	Satisfactory	Satisfactory	Satisfactory	Overexposure	GTR	As planned
37	CPA region	Prominent	Satisfactory	Satisfactory	Satisfactory	Reasonable	GTR	As planned
38	CPA region	Prominent	Satisfactory	Satisfactory	Satisfactory	Reasonable	GTR	As planned
39	CPA region	Prominent	Satisfactory	Satisfactory	Satisfactory	Reasonable	GTR	As planned
40	Sphenoid ridge	Prominent	Satisfactory	Satisfactory	Satisfactory	Reasonable	GTR	As planned
41	Petroclival region	Prominent	Satisfactory	Satisfactory	Satisfactory	Reasonable	GTR	As planned
42	Infra-temporal fossa	Prominent	Satisfactory	Satisfactory	Satisfactory	Reasonable	GTR	As planned
43	CPA region	Prominent	Satisfactory	Satisfactory	Satisfactory	Reasonable	GTR	As planned
44	Petroclival region	Prominent	Satisfactory	Satisfactory	Satisfactory	Reasonable	GTR	As planned
45	CPA region	Prominent	Satisfactory	Satisfactory	Satisfactory	Reasonable	GTR	As planned
46	Infra-temporal fossa	Prominent	Satisfactory	Satisfactory	Satisfactory	Reasonable	GTR	As planned
47	Infra-temporal fossa	Prominent	Satisfactory	Satisfactory	Satisfactory	Reasonable	GTR	As planned

*No., number; GTR, gross total resection; STR, sub-total resection; PR, partial resection; CPA, cerebello-pontine angle; ICA, internal carotid artery*.

### Representative Cases

**Case 1:** A 32-year-old man with a recurrent C2 schwannoma (Supplementary Video 1).

**Case 27:** A 52-year-old man with a recurrent spindle cell tumor of the left cranio-orbital communication (Supplementary Video 2).

**Case 46:** A 57-year-old woman with a lobular malignant tumor of the left infratemporal fossa (Supplementary Video 3).

## Discussion

Multimodal image fusion technology benefits pre-operative surgical planning and resection in all types of skull base tumor cases (16–18). In cases in which the retrosigmoid approach as used, we made the incision based on the multimodal fusion image, which clearly displayed the spatial relationship of the tumor, transverse sinus, and sigmoid sinus. The bone flap could also be precisely designed in all 47 cases, except for 2 cases of overexposure. In both cases (1 case of an olfactory groove meningioma and 1 case of an acoustic schwannoma), unexpected intraoperative brain swelling occurred and hampered the surgical site exposure. Overall, the main arteries and veins were consistent with the intraoperative findings. We evaluated the application value of every case involved in our study individually based on the evaluation criteria. Forty cases were prominent, which was substantial for the resection procedure, and 7 cases were supportive. In 7 supportive cases, 1 case was a cholesteatoma in the CPA region, 2 cases were meningiomas of the planum sphenoidale, and 1 case was a meningioma of the sphenoid ridge. Those 4 cases were both clearly located with no bone deconstruction, and no significant nerves or arteries were adhered to the tumor; this could be seen on the two-dimensional MR or CT scan. In 1 acoustic tumor case, the volume of the tumor was small and the auditory canal was not penetrated, so total resection was possible based on only the pre-operative MR image. In 1 case of an occipital-cervical schwannoma that was not adhered to the vertebral artery, the tumor was fully visible in the outer dura mater on the MR scan. Except 1 case of a squamous cell carcinoma of the left external auditory canal with petrosal bone and temporal base infiltration, the partial resection plan could only be determined intraoperatively due to the inability to fully observe the degree of adhesion between the tumor, internal cortical artery, and petrosal segment pre-operatively. In that case, a supportive evaluation was given. However, in MR T2-FLAIR sequence, the moisture content could be demonstrated so the degree of hardness could be primarily evaluated (19). In that case, a primary comprehensive MR image is still needed.

The total resection rate was 78.7%, which was mildly higher than that previously reported without the multimodal image fusion technique. In most previous cases, the number of intracranial and extracranial communication tumors and malignant tumors was lower than that of non-intracranial and extracranial communication tumors and benign tumors. Our study showed a similar result. Generally, when tumors have a high degree of adhesion with major blood vessels, the total resection rate is low. There were two post-operative cases shifted to radiotherapy department due to partial resection which were petroclival region meningioma for one case and infra-temporal fossa meningioma for one case. Both of those two cases were occurred unexpected intraoperative bleeding due to high adhesion.

The mean operative time was mildly shorter than that in some previous reports in which the multimodal image fusion technique were not applied (20–22). The operative time in the posterior skull base in the present study was shorter than that in other case reports (23, 24). In one study, the original craniotomy technique of the posterior sigmoid sinus keyhole approach was used by the surgeon's team, and the new method kept the craniotomy time within 22–25 min (25).

In the current study, there were 3 cases of intracranial infection including 2 cases of middle skull base tumors. Extracranial communication and malignant tumors were mostly accompanied by bony defects and severe adhesion (26, 27); thus, the long operative time and difficult resection would increase the complication rate. Post-operative complications of the posterior skull base were mainly facial paralysis, most of which improved after symptomatic treatment. Two patients with dysphagia underwent post-operative tracheotomy, but their swallowing function recovered after discharge.

Skull base reconstruction is the key to preventing cerebral fluid leakage and intracranial infection, and is one of the important factors for successful intracranial base resection (28, 29). In this study, we used the multimodal image fusion technique to evaluate the reconstruction plan, and bone defects were fully visible and titanium plates for reconstruction were formed pre-operatively. Only 6 cases of cerebrospinal fluid leakage occurred post-operatively, mainly due to severe damage of the skull base fracture and failure to repair damaged meninges intraoperatively. Skull base reconstruction should be fully comprehensible based on the pre-operative incision design. Intraoperative separation of tumors adhered to the meninges should be performed carefully and gently to minimize damage to their structural integrity. During intraoperative resection of the tumor, attention should be paid to preserving the meningeal residual margin of the skull base and not to overexcising the meningeal residual margin by emphasizing total tumor resection. There should be enough cap-shaped aponeurosis and periosteum for dural repair. For skull defects <4 cm in diameter, the dural was closely repaired using a pedicled bone valve or cap aponeurosis without skull reconstruction. Cranial reconstruction should be considered for skull defects ≥4 cm in diameter. If cerebral fluid leakage occurs after surgery, lumbar cistern drainage is feasible; if cerebral fluid leakage still exists after 1 month of conservative operation, a secondary operation to fix the leakage is needed (30).

At present, multimodal image fusion technology still has some limitations, as it is difficult to reconstruct tiny vessels. However, 3D reconstruction has high requirements for image inspection, such as a scanning layer thickness >2 mm, which may lead to a roughly reconstructed image and affect the reference value.

## Conclusions

The multimodal image fusion technique can achieve satisfactory results and should be strongly recommended for skull base tumor surgery. Derived from this technique, mixed virtual reality technology has broad prospects for assisted surgery of skull base tumors in the future. A follow-up study is ongoing by our team.

## Data Availability Statement

The original contributions presented in the study are included in the article/Supplementary Material, further inquiries can be directed to the corresponding author.

## Ethics Statement

The studies involving human participants were reviewed and approved by Medical Ethics Committee of Zhuhai People's Hospital (Number: ZY.no20201001B06011231). The patients/participants provided their written informed consent to participate in this study. Written informed consent was obtained from the individual(s) for the publication of any potentially identifiable images or data included in this article.

## Author Contributions

GC and Z-hJ contributed to the study concept and design. J-yL and H-dC contributed to the acquisition of data. YL and K-hW contributed to the analysis and interpretation of data. Z-hJ contributed to the drafting of the manuscript. All authors read and approved the final manuscript.

## Funding

This work was supported by Soochow Key Health Talents Project of Jiangsu Province, 2014, GC and Zhuhai People's Hospital Scientific Research Initiation Project No. 2021KYQD-02 to GC.

## Conflict of Interest

The authors declare that the research was conducted in the absence of any commercial or financial relationships that could be construed as a potential conflict of interest.

## Publisher's Note

All claims expressed in this article are solely those of the authors and do not necessarily represent those of their affiliated organizations, or those of the publisher, the editors and the reviewers. Any product that may be evaluated in this article, or claim that may be made by its manufacturer, is not guaranteed or endorsed by the publisher.
